# The Diagnosis and Treatment of a Rare Maxillary Plasmacytoma: a Case Report

**DOI:** 10.30476/DENTJODS.2019.77839.

**Published:** 2020-09

**Authors:** Amir Mansour Shirani, Atefeh Akhani, Vahid Esfahanian, Shahab Etemadi, Mohammad Reza Mohajeri

**Affiliations:** 1 Dept. of Oral Medicine, School of Dentistry, Isfahan (Khorasgan) Branch, Islamic Azad University, Isfahan, Iran; 2 Oral Medicine Specialist School of Dentistry, Isfahan (Khorasgan) Branch, Islamic Azad University, Isfahan, Iran; 3 Dept. of Periodontology, School of Dentistry, Isfahan (Khorasgan) Branch, Islamic Azad University, Isfahan, Iran; 4 Dept. of Oral and Maxillofacial Radiology, School of Dentistry, Isfahan (Khorasgan) Branch, Islamic Azad University, Isfahan, Iran; 5 Clinical Pathologist, Isfahan, Iran

**Keywords:** Plasmacytoma, Multiple myeloma, Maxillary bone, Radiotherapy, IHC staining

## Abstract

The jaw plasmacytoma is a very rare condition, which its diagnosis is difficult in clinical routine. Up to now, less than 60 cases of jaw plasmacytoma have been reported in the literature. In the present case report, we reported a rare case of jaw plasmacytoma in a 42-year-old female, which was misdiagnosed with dental granuloma and abscess. The diagnosis of plasmacytoma was done by immunohistochemistry (IHC) evaluation following a cone beam computed tomography (CBCT) assessment. The patient was treated with radiotherapy and is disease free after 2 years.

## Introduction

Solitary plasmacytoma is one of the rarest diseases, which is categorized into solitary bone plasmacytoma and extramedullary solitary plasmacytoma [ 1 , 10 ] . The disease mainly affects the axial skeleton comprising the vertebrae, ribs, and pelvis [ 4 ]. Solitary bone plasmacytoma of the jaw is a very infrequent disease with less than 60 reported cases in the literature. It presents more commonly among males ages 50-80 years [ 11 , 12 ] . Here, we present a 42-year-old female who presented with jaw solitary bone plasmacytoma, which was misdiagnosed with dental granuloma and abscess. 

## Case Presentation

**Figure 1:**
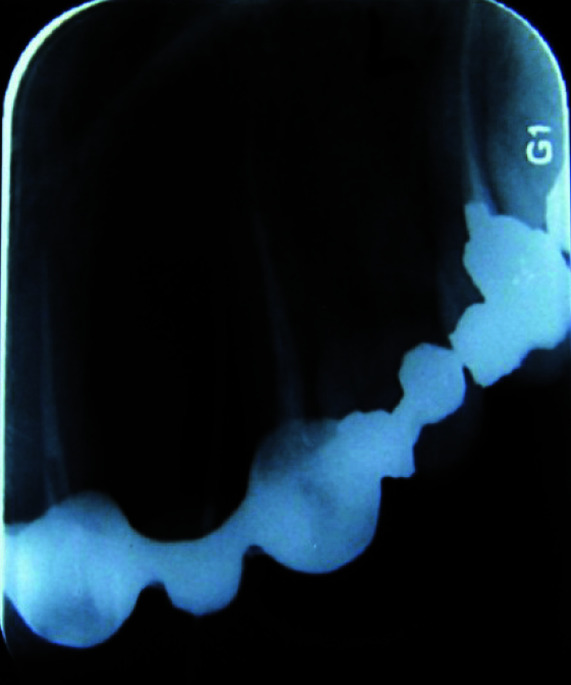
The first radiographic view of the lesion in the early stage.

A 42-year-old woman was admitted to an oral and maxillofacial surgery clinic for the evaluation and treatment of a large radiolucent lesion with a light aching pain in the region of the left maxilla. The patient presented with a past medical history of a swelling in her left zygomatic area treated by antibiotic. She also had a history of an erythema and radiolucent lesion of gingival tissue in the maxillary posterior teeth region around the porcelain fused to the metal bridge of teeth 21-23 Figure 1). The pathological evaluation of the lesion following biopsy had shown dental granuloma with chronic abscess. After ccomplete curettage of the bony lesion, hard and soft tissue augmentation had been performed to repair severe bone loss at bridge area. Root canal therapy (RCT) of the teeth number 24-26 had also been done to control pain in the posterior maxilla; however, the region had remained painful.

**Figure 2:**
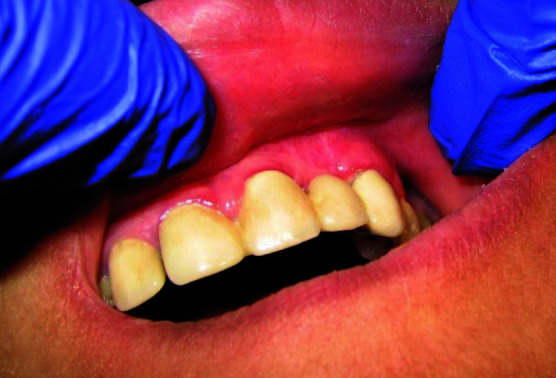
Mild gingiva erythema in bonny tumor location without any expansion.

**Figure 3:**
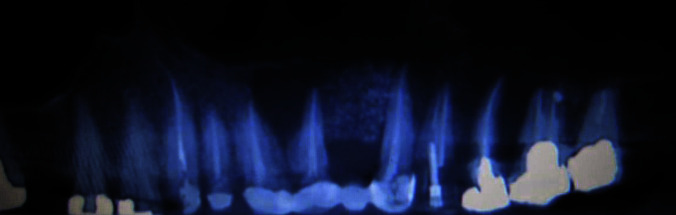
CBCT (Cone-Beam Computed Tomography) view: large radiolucent lesion between the teeth 11- 27

Finally, a large ill-defined radiolucent lesion of the left maxilla had been observed in the panoramic view. The clinical examination showed light erythema on left maxillary gingiva without any bony expansion. Grade 3 mobility was also found in the tooth number 25 (Figure 2). Routine hematological and biochemical indices (complete blood count differential and the immunoglobulins) were within normal reference range except for ESR (27 mg/dl). The outcome of the cone beam computed tomography (CBCT) revealed a wide spread ill-defined radiolucent lesion between the teeth numbers 11 to 27, with severe destruction of buccal and palatal bones and loss of sinus and nasal floor in some areas; while, there was no sign of root resorption. Floating in air appearance of the tooth number 25 was also seen (Figures 3- [Fig JDS-21-239-g004.tif] 5). The periapical radiography showed an ill-defined radiolucent lesion around the teeth number 24 and 25 (Figure 6). After flap preparation and under local anesthesia, a biopsy was taken and sent for histopathological evaluation. A granulation-like tissue and severe bonny destruction was observed in the area. The tooth number 25 was also extracted because of severe mobility. 

A diagnosis of plasmacytoma was confirmed following histopathological analysis (Figure 7-[Fig JDS-21-239-g008.tif] [Fig JDS-21-239-g009.tif] 10). A whole-body bone scanning showed no abnormality and bone marrow cells were normal following bone marrow biopsy. The urine’s Bence Jones protein was negative and there were no symptoms of multiple myeloma in the blood tests. The patient was treated with two-field conventional radiotherapy (total field dose 2500 Gy). The mobility of the tooth number 26 was decreased gradually and gingival erythema was resolved. Over two years of follow up, dryness of mouth was controlled and there was no sign of recurrence.

**Figure 4 JDS-21-239-g004.tif:**
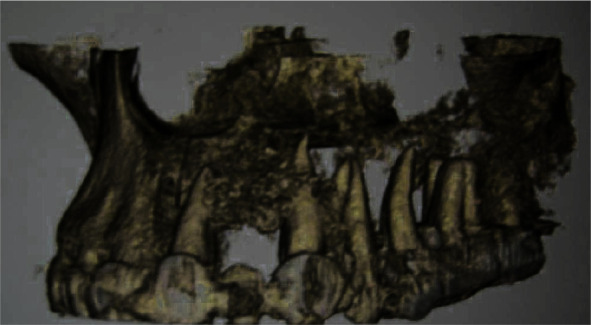
CBCT (Cone-Beam Computed Tomography) view: large radiolucent lesion between the teeth 11 - 27

**Figure 5:**
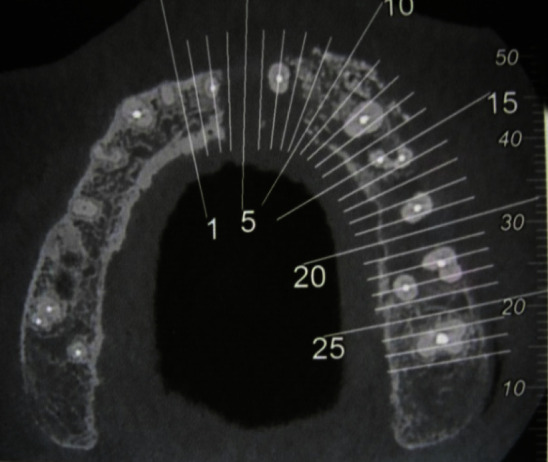
CBCT (Cone-Beam Computed Tomography) view: large radiolucent lesion between the teeth 11 - 27

**Figure 6:**
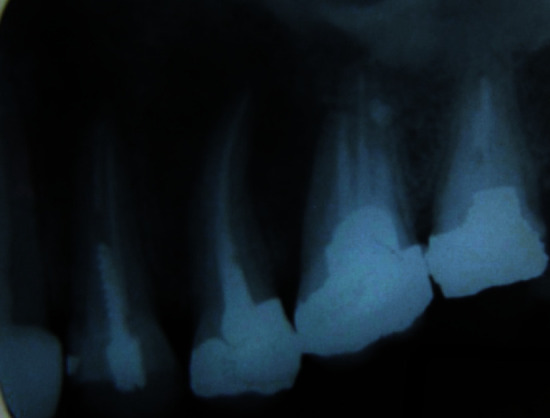
The periapical radiography view: the severe damage was seen in the tooth 24 and 25 region.

**Figure 7:**
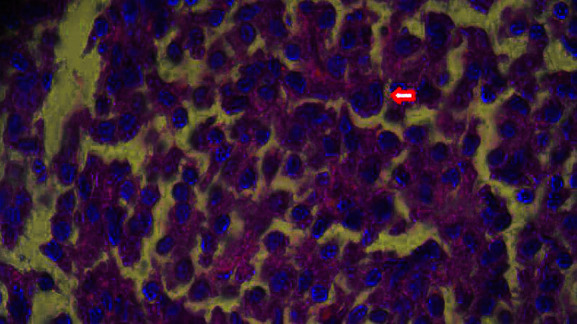
Infiltration of neoplastic large binucleated plasma cell (hematoxylin and eosin, original magnification 400×).

**Figure 8 JDS-21-239-g008.tif:**
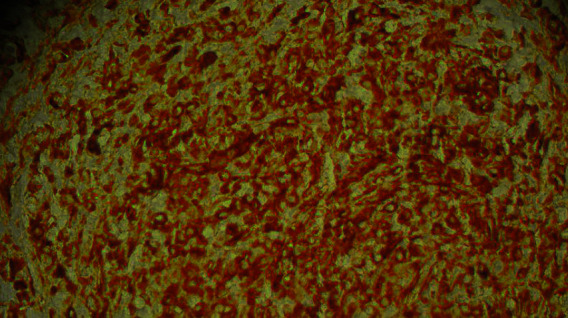
Immunohistochemical staining showing immunop-ositivity for Vimentin (400×).

**Figure 9 JDS-21-239-g009.tif:**
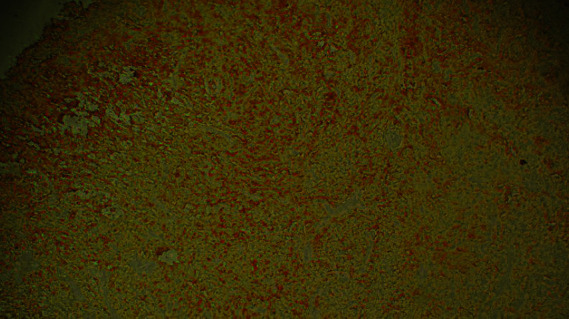
Immunohistochemical staining showing immunop-ositivity for Kappa (100×).

**Figure 10:**
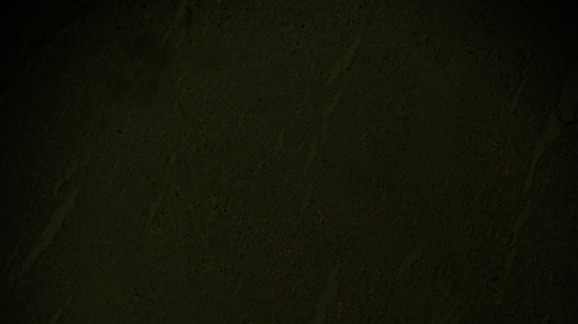
Immunohistochemical staining showing immunon-egativity for Lambda (100×).

## Discussion

Maxillary plasmacytoma is a very rare clinical condition, which its incidence rate increases with age. Some previous reports have implied that there were no differences in the prevalence of Maxillary plasmacytoma between males and females [ 4 , 19 , 21 ] . However, it has also been suggested that the prevalence of the disease in men is twice than that in women [ 11 ]. Clinical signs and symptoms include local pain, bone fractures, and dysfunction of the bone. In the physical examination, the presence of progressive swelling in the jawbone or the involvement of oral mucosa and other soft tissues may be observed [ 13 , 14 ] . 

However, in the current case only small erythema of gingiva without any swelling was observed. It is difficult to diagnose the disease in early stages because of the non-specificity of its symptoms. In this case, the patient has been undergone unnecessary bone augmentation and RCT because of the misdiagnosis of the disease. The current diagnostic criteria of solitary bone plasmacytoma are defined as the presence of an isolated region of bone destruction due to clonal plasma cells, a bone marrow examination morphologically normal, or having a very low clonal plasma cell infiltration (less than 10%). Moreover, the absence of other osteolytic lesions or the involvement of other soft tissues, negative history of anemia and hypercalcemia or absence of kidney failure and low serum or urine level of monoclonal protein are considered as other diagnostic criteria [ 18 ]. Though, the current patient fulfilled the diagnostic criteria, the ultimate diagnosis was made after one year considering the low number of malignant cells in pathological evaluation, absence of specific laboratory, and clinical markers. In hematological tests, ESR was the only factor, which was out of normal range. We suppose that ESR can be considered as a possible useful maker for the diagnosis of the malignancy.

Allegra et al. [ 19 ] also introduced a 43-year-old Chinese male patient with a maxillary swelling, which was initially diagnosed as an abscess, but later pathological assessments revealed solitary plasmacytoma. In a 62-year-old case reported by Kamal et al. [ 20 ], a short-term non-painful swelling in the right lower mandibular region was observed. Although the radiological findings were inconclusive, a pathologic examination revealed a solitary plasmacytoma. In the case, the bonny destruction was not observed in panoramic radiography; however, it was seen clearly in CBCT examination. 

A systematic review [ 2 ] presented 50 case reports on patients with a solitary plasmacytoma of the jaw. The results of the study showed that the tumor usually appears as a single osteolytic lesion, without plasmacytosis in the bone marrow. It was also reported that the prognosis of the disease is very poor, and about half of studied cases progress to multiple myeloma. As a result, the early diagnosis and treatment of the tumor can prevent its progression toward multiple myeloma [ 2 ]. Surprisingly, Cioranu et al. [ 21 ] reported a patient with multiple solitary plasmacytoma in a 14-years period evaluation without progression of multiple myeloma. It has been suggested that early treatment of the disease reduces the local complications of the tumor [ 2 , 22 ]. In some cases, symptoms may be last from 1 month to 1.5 years (average 9 months) prior to the diagnosis [ 13 - 14 ]. In this case, the time between first complication and final diagnosis was about 14 months.

A 64-year-old male with a tumor in the left mandibular angle with extension to the parotid region of the same side was introduced by Rodriguez-Caballero et al. [ 22 ]. Different radiological assessments revealed an osteolytic lesion in this area and confirmed by the pathologic evaluation as solitary plasmacytoma. The outcome of radiotherapy for plasmacytoma in the patient was satisfactory as was for our case. 

## Conclusion

Plasmacytoma is a rare local form of multiple myeloma and it may mimic signs of dental infection in clinical and pathologic evaluation. CBCT and IHC tests are very useful for the diagnosis of the malignancy. ESR test can also be considered as a helpful laboratory index for the diagnosis of the disease. Further investigations are recommended to endorse these findings. 
